# Dark accelerates dissolved inorganic phosphorus release of high-density cyanobacteria

**DOI:** 10.1371/journal.pone.0243582

**Published:** 2020-12-22

**Authors:** Mengmeng Wang, Huifen Zhang, Menggaoshan Chen, Liuyan Yang, Yichen Yang

**Affiliations:** 1 State Key Laboratory of Pollution Control and Research Reuse, School of the Environment, Nanjing University, Nanjing, China; 2 Department of Agricultural and Biological Engineering, University of Florida, Gainesville, FL, United States of America; Cawthron Institute, NEW ZEALAND

## Abstract

Bloom-forming cyanobacteria dramatically influence nutrient cycling in eutrophic freshwater lakes. The phosphorus (P) assimilation and release of bloom-forming cyanobacteria significantly may also affect the phosphorus source and amounts in water. To understand the phosphorus release process of bloom-forming cyanobacteria below the accumulated surface and sedimentary bloom-forming cyanobacteria, the degradation of bloom-forming cyanobacteria dominated by *Microcystis* spp. at different cell density in the dark was investigated over a 25-day microcosm experiment. The dissolved inorganic phosphorus (DIP) and dissolved total phosphorus (DTP) contents increased with the increment of cyanobacterial density, and the dark status markedly increased the proportion of DIP in water during the decline period of bloom-forming cyanobacteria. Meanwhile, the process of cyanobacterial apoptosis accompanied by the changes of malondialdehyde (MDA) and phosphatase (AKP) contents, and the increases of superoxide dismutase (SOD) and catalase (CAT) activities of cyanobacteria in the dark, especially in low-density groups (5.23×10^8^ cells L^-1^), which further affect the physicochemical water parameters. Moreover, the DIP release from high-density cyanobacteria (7.86×10^7^ cells L^-1^~5.23×10^8^ cells L^-1^) resulted from the relative abundance of organophosphorus degrading bacteria in the dark. Therefore, the fast decay of cyanobacteria in the dark could accelerate DIP release, the high DIP release amount from accumulated bloom-cyanobacteria provide adequate P quickly for the sustained growth of cyanobacteria.

## 1 Introduction

Phosphorus (P) is a vital biogenic element [1–4], and its biogeochemical cycle plays crucial roles in freshwater ecosystems [5, 6]. Excessive P can eutrophicate freshwater bodies, which inevitably leads to harmful algal blooms, especially cyanobacterial bloom [4, 7, 8]. Therefore, phosphorus is regarded as the vital index of lake trophic status evaluation [9, 10]. In the past decades, in China, cyanobacterial bloom, as the most severe water quality disaster, broke out continually in eutrophic shallow lakes due to exogenous P. There is no doubt that the restoration of eutrophic lakes is extremely urgent [10, 11]. Our survey in recent years showed that cyanobacterial blooms transferred gradually from the northern area to the western area in Lake Taihu, accompanied by the P increase in the western lake area. We speculate that the P release from accumulated bloom-cyanobacteria aggravates P content in the water of Lake Taihu.

However, numerous early studies mainly focused on the P uptaking and utilization by cyanobacteria, relatively little attention has been paid to the P release from bloom-forming cyanobacteria, especially in the dark. Chen et al. [12] found that the dissolved P released by aerobic degradation of *Microcystis* accounted for 53% of the total P in water. Water extractable P concentrations increased with the time during the degradation of *Microcystis aeruginosa* [13]. It was reported that the colloidal P concentration was about five times higher than those at the beginning when the cyanobacteria were decomposed [14]. Microcosm study also showed that the high dissolved P concentration in the water column of Meiliang Bay of Lake Taihu resulted from the decomposition of cyanobacteria during the outbreak of cyanobacterial bloom [4]. The determination of an in-situ enclosure experiment in Lake Dianchi showed that the decline period of cyanobacterial bloom was accompanied by P release [11]. Therefore, P release from phytoplankton, such as bloom-forming cyanobacteria, plays an essential role in the biogeochemical cycle of P in freshwater systems. Among these studies, some of them are from the P release of healthy cyanobacterial metabolism, and some P releases are conducted in natural decay. Given the dramatic differences in P release mode, the determination of P release from bloom-forming cyanobacteria at high cell density and dark status simulates the P release of cyanobacteria below the accumulated surface and sedimentary bloom-forming cyanobacteria may be more helpful to our understanding on the P cycles in the aquatic environment.

Lake Taihu is the third-largest freshwater lake in China and has suffered from cyanobacterial blooms in the past few decades [4, 15, 16]. The total phosphorus (TP) concentration of Lake Taihu increased significantly along with more than 1000 km^2^ cyanobacterial bloom in 2016–2017, which caused considerable threats to the water quality and ecosystem [10]. Meiliang Bay, located in the northern areas of Lake Taihu, is one of the areas that suffered from massive cyanobacterial bloom in summer. Bloom-forming cyanobacteria accumulate along the coast on account of southeast wind at high temperatures. The survey found that the topical accumulating thickness of cyanobacteria bloom could reach up to a few centimeters or more under certain meteorological conditions. If P release from the anaerobic decomposition of highly intensive cyanobacteria could sharply increase dissolved P, the extensive bloom-forming cyanobacteria should be a factor in the spatial difference of P distribution of water in Lake Taihu. The overall objective of this work is to investigate the effect of the dark caused by cyanobacteria accumulation on P release of the high-density cyanobacteria and analyze the relationships between P release with physiological indexes of cyanobacteria, water quality, and attached bacterial community to provide a theoretical basis for cyanobacterial control.

## 2 Materials and methods

### 2.1 Study area

Meiliang Bay has an area of 122 km^2^ and locates in the northern part of Lake Taihu. It is a typical cyanobacterium dominated lake region [17, 18]. Cyanobacterial blooms frequently occur in this area, and *Microcystis* spp. are the dominant species in cyanobacterial blooms [17]. Cyanobacteria, 91.9% of which is *Microcystis*, constitute up to 32.7% of the total phytoplankton biomass in Meiliang Bay [4].

The environmental parameters of the sampling point were measured as shown in Table 1. The water temperature (T) was about 28.6 ^0^C, while the pH value was 8.22, and the DO concentration was 3.48 mg L^-1^ in the area of cyanobacterial accumulation. Other parameters such as electroconductibility (EC), salinity (Salt), oxidation-reduction potential (ORP), and total dissolved solids (TDS) values remained relatively constant (Table 1). Different forms of P such as total phosphorus (TP), total dissolved phosphorus (TDP), dissolved inorganic phosphorus (DIP) were also determined in the sampling lake water.

**Table 1:** The physicochemical parameters of the water body in the sampling point.

Parameters	Value	Parameters	Value
Chl-a (μg mL^-1^)	4.32	Temperature (°C)	28.60
TP (mg L^-1^)	1.02	pH	8.22
DIP (mg L^-1^)	0.04	TDS (mg L^-1^)	302.00
TDP (mg L^-1^)	0.34	ORP (mV)	-35.20
DO (mg L^-1^)	3.48	EC (μS cm^-1^)	510.00
TN (mg L^-1^)	6.93	Salt (ppt)	0.47

### 2.2 Preparation and setting of the simulation experiment

The fresh bloom-forming cyanobacteria were collected from Meiliang Bay (31.536302N, 120.173059E) in August 2019, where cyanobacteria accumulated. The microscopic results showed that the proportion of *Microcystis* was 99.81%, and zooplankton was rarely found. The collected cyanobacteria were diluted into three densities (5.23×10^8^ cells L^-1^, 3.10×10^8^ cells L^-1^, 7.86×10^7^ cells L^-1^) with the in-situ lake water, which was filtered by filter membrane (0.22 μm). The cyanobacterial sample was sealed into 500 mL glass bottles after mixing evenly. In order to simulate the dark condition of local accumulation of cyanobacteria. Each accumulated density was divided into the dark group (in the dark) and illumination group (5000 lx, 12 h/12 h light/dark) to simulate the effect of the dark on the accumulated cyanobacteria. The groups were denoted as high (HD), medium (MD), and low (LD) accumulated cyanobacterial densities in the dark and high (HI), medium (MI), and low (LD) accumulated cyanobacterial densities with illumination. Finally, all the bottles were placed in an incubator with 28°C. Three replicates were conducted in each treatment group, and the experiment was conducted for 25 d. The indexes of water quality were measured each 5 d.

### 2.3 Analytical methods

#### 2.3.1 Determination of Chl-a content and cyanobacterial density

Aliquots (10 mL) were collected from the bottles by a syringe after shaking well and refrigerated for Chl-a analysis. These samples were filtered, ground, extracted by 90% acetone, and centrifuged. The absorbance of Chl-a was determined by a UV-1800 spectrophotometer (Shimadzu, Japan) [19]. The cyanobacterial cells were counted under a microscope after being stained with Lugol’s solution.

Cyanobacterial net mortality = (*C*_0_-*C*_t_)/*C*_0_×100%
where *C*_0_ is the initial cyanobacterial density;
*C*_t_ is the cyanobacterial density at time t (d).

#### 2.3.2 Physiological parameters of cyanobacteria

The cyanobacterial water sample (3.0 mL) was centrifuged at 4°C at 12,000 rpm for 5 min. Then the precipitation was retained, and the supernatant was replaced by 3.0 mL PBS buffer (0.05 mol L^-1^, pH 6.8). The supernatant was used for the detection of AKP in extracellular cells. After the mixture was gently shaken, it was frozen at -80°C and thawed at room temperature four times. The clear supernatant was taken for protein determination using the bicinchoninic acid (BCA) protein assay kit (Nanjing Jiancheng Bioengineering Inc., Nanjing, China) after centrifugation at 12,000 rpm for 5 min. Total superoxide dismutase (SOD) activity, malondialdehyde (MDA) levels, Catalase (CAT) activity, and Alkaline phosphatase (AKP) activity were measured using SOD, MDA, CAT, AKP assay kits (Nanjing Jiancheng Bioengineering Inc., Nanjing, China) in strict accordance with kit requirements.

#### 2.3.3 Determination of water quality parameters

The water quality parameters (T, DO, pH, ORP, EC, Salt, and TDS) were detected by HACH HQ30-D (Hach, Loveland, CO, USA) during the experimental periods. Total nitrogen (TN), total phosphorus (TP), dissolved total phosphorus (DTP), dissolved inorganic phosphorus (DIP), ammonia nitrogen (NH_3_-N), nitrite-nitrogen (NO_2_^-^-N), and nitrate-nitrogen (NO_3_^-^-N) were analyzed according to standard methods [20].

#### 2.3.4 Isolation, PCR amplification of DNA sequencing in bacteria

The cyanobacterial water sample (20 mL) was filtered with a 150 μm-diameter polycarbonate filter membrane to remove the larger plankton, and then the filtrate was filtered with a 3 μm polycarbonate filter membrane. The attached bacteria of cyanobacteria were intercepted by filtering the filtrate with a 0.22 μm polycarbonate filter membrane.

The V3-V4 region of the 16S rRNA gene was sequenced using 341F (5'-CCTACGGGNGGCWGCAG-3') and 805R (5'-GACTACHVGGGTATCTAATCC-3') as universal primers of the 16S rRNA gene. After purification with Axy Prep DNA Gel Extraction Kit (Axgen, USA), sequencing was completed at Shanghai Sangon Biotech Biotechnology Co., Ltd. After sequencing, the raw data were spliced and filtered to obtain clean data for subsequent analysis. Then the operational taxonomic units were classified, and the species classification (RDP classifier) was carried out. Each sequence categories were determined at the phylum, class, and genus levels, and the number and relative abundance of sequences within each category were counted.

### 2.4 Data treatment and statistical analysis

Analysis of statistical significance and the Pearson’s correlation was carried out with SPSS software (SPSS Inc., Chicago, IL, USA). Bar charts and line charts were mapped using OriginPro 2018 software (Origin Lab, Northampton, MA, USA). The microbial diversity was measured by analyzing the alpha diversities, as determined using the QIIME pipeline and based on the OTUs. For alpha diversity, Chao1 richness, and the Shannon and Simpson diversity indices were calculated using QIIME [21].

## 3. Results and discussion

### 3.1 Dark promotes DIP release of high-density cyanobacteria

**Fig 1:**
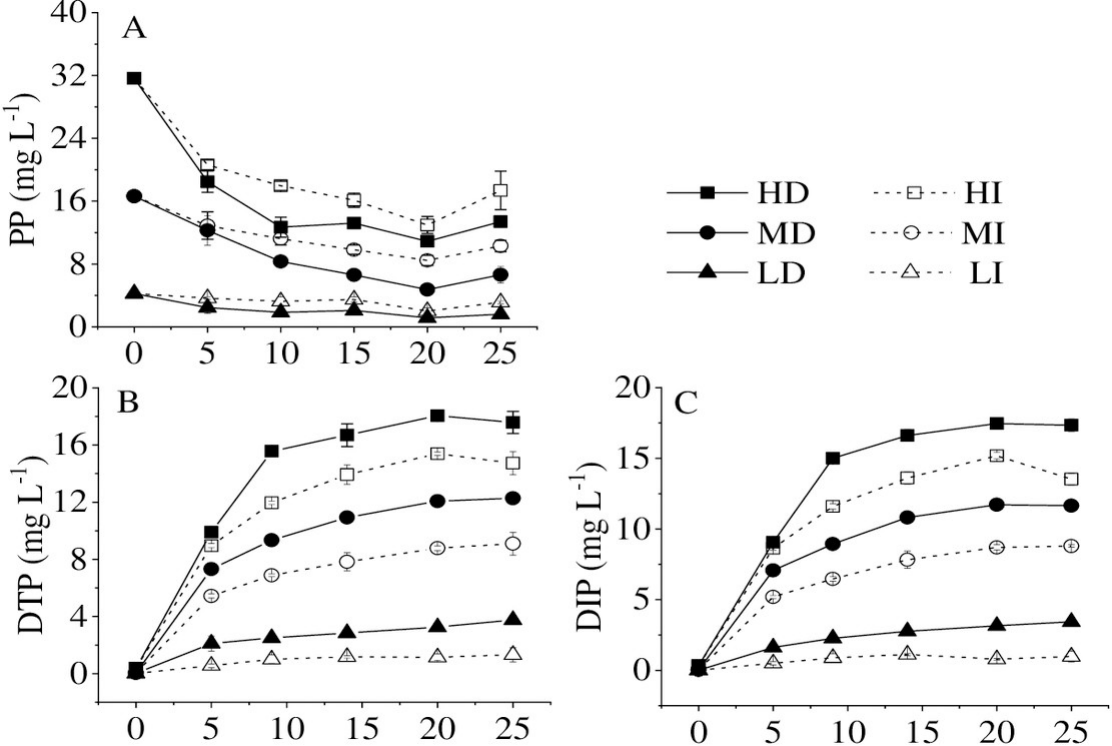
Changes of dissolved total phosphorus (DTP) (A), particle phosphorus (PP) (B), and dissolved inorganic phosphorus (DIP) (C) in different treatments at high (HD), medium (MD) and low (LD) accumulated cyanobacterial densities in the dark and high (HI), medium (MI) and low (LD) accumulated cyanobacterial densities with illumination.

To explore the effect of dark on the phosphorus release from high-density cyanobacteria, DTP, DIP, and particle phosphorus (PP) concentrations in water were determined during the microcosm experiment. DTP and DIP concentrations increased dramatically, accompanied by the declination of bloom-forming cyanobacteria at different cyanobacterial accumulated densities and different illumination conditions, while the PP concentration decreased and reached a steady level later (Fig 1). A few studies showed that bloom-forming cyanobacteria could release P into the water, which was one of the most critical processes that affect the biogeochemical cycle of P [22–24]. Cao et al. [25] and Zhang et al. [11] both reported that P could be released from the dead cyanobacteria into the water after the cyanobacteria began to decline, which is consistent with our results. Cyanobacteria released P rapidly during the first 5-days, the release rates were 1.90, 1.71,1.45, 1.08, 0.42, and 0.11 mg L^-1^ d^-1^ in HD, HI, MD MI, LD, and LI, respectively. After 20 d of degradation, the amounts of DTP and DIP reached the maximum level. This could be explained in two aspects. Physical leaching from the fresh cyanobacteria may contribute to part of DTP and DIP [26, 27]. Besides, complete degradation of cyanobacteria in a short period was previously observed in the field [4, 11, 14], which accorded with our simulation results (i.e., up to 19.49%, 24.87%, 22.84%, 21.80%, 55.04%, 28.73%) of cyanobacterial amounts decrease in the HD, HI, MD, MI, LD, LI treatments, respectively during the first 5-days.

In this study, the released amounts of DTP and DIP were always higher in the dark than in the illumination at the same initial cyanobacterial accumulated density during the experiments. In contrast, the PP concentration was lower in the dark than that in the illumination. The first, some living cells left in the illumination, could re-assimilate the P for their growth [28, 29]. Especially at very low density, cyanobacteria could keep alive for a long time along with P uptake and release reached steady state [28]. The second, an anoxic or anaerobic environment in the dark, is not conducive to cyanobacterial growth [11, 23]. Furthermore, with the increase of cyanobacterial accumulated density, the release amounts of DTP and DIP increased under the same light condition (Fig 1B and 1C). The results were the same as the finding reported by Chuai et al. [4]. Therefore, if more cyanobacteria accumulate in an area or deposit to the bottom, amounts of dissolved inorganic phosphorus will be released into the water, which may be a crucial factor for the persistent outbreak of cyanobacterial bloom in a lake.

When bloom-forming cyanobacteria were in the process of dying, the ratios of DTP/TP and DIP/TP gradually increased and reached the highest value in the water body at the 20^th^ d. DIP/DTP ratios exceeded 90% in the whole experiment in all treatments. Therefore, bloom-forming cyanobacterial accumulation promotes dissolved inorganic phosphorus release, which is consistent with Zhang et al. [11] that the primary P forms from cyanobacteria released into the water were DIP and organic phosphorus (OP), especially DIP. Bai et al. [30] also observed that OP could be decomposed into active soluble phosphorus by corresponding microorganisms under anaerobic, anoxic, and aerobic conditions. It was reported that the DIP release was determined by the transformation of organic P in the dead cyanobacterial cells and the breakage of the mucilage sheath [11]. The result from Zhe et al. [31] showed that the cyanobacterial declination could contribute to a rapid increase in bacterial populations, and then the dissolved organic matter released by decomposed cyanobacteria would be quickly consumed and re-mineralized by bacteria [32]. At the same time, the capsular polysaccharide content per cell decreased during the *Microcystis* bloom decline period. Meanwhile, the sheath structure of *Microcystis* colonies changed from the complete to the broken [33], which significantly accelerated DIP release from cyanobacteria.

**Fig 2:**
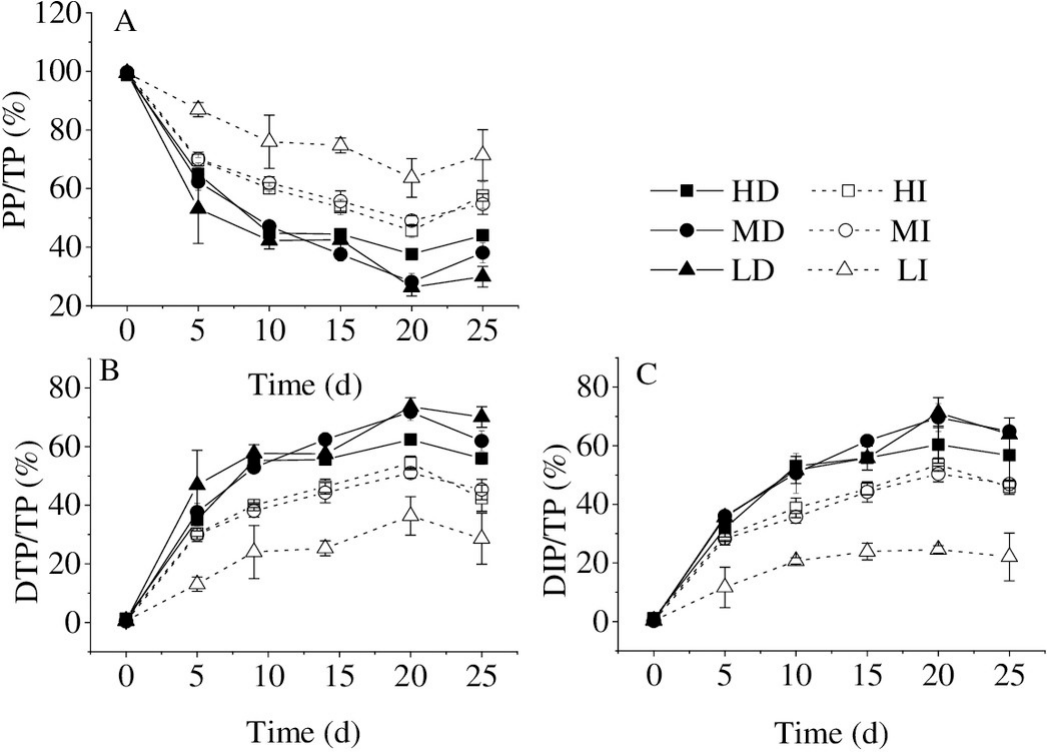
The phosphorus forms percentages of PP/TP (A), DTP/TP (B), and DIP/TP (C) in different treatments. Values given are the mean ± SD of three replicate measurements.

Regardless of the initial accumulated cyanobacterial density, the ratios of DTP/TP and DIP/TP were higher in the dark than in the illumination (Fig 2B and 2C). The ratio of PP/TP was higher in the illumination than in the dark, irrespective of the initial cyanobacterial accumulated density (Fig 2A). In the illumination, the higher accumulated bloom-forming cyanobacterial density was, the lower the ratio of PP/TP was. Additionally, the ratio of PP/TP was much higher at low cyanobacterial density than at high and medium cyanobacterial densities. In the dark, the ratio of PP/TP decreased with time at the high, intermediate, and low accumulated cyanobacterial densities, while there was no difference among the different density groups (Fig 2A). The first, some living cells left could re-assimilate the P for their growth [28, 29], especially in low cyanobacterial density and illumination treatment groups. The second, the cyanobacterial cells are encapsulated by an extracellular polysaccharide [34], which decomposed along with the cyanobacterial decay. The DIP release from cyanobacteria in the illumination may be absorbed again by cyanobacteria. In a word, reduction of consumption and enhancement of decomposition are the main reasons for the higher DIP/TP ratio in the dark. Thus, the transformation from organic phosphorus to inorganic phosphorus is the primary phosphorus cycling process on the opaque layer of cyanobacteria bloom accumulation.

The area of cyanobacterial blooms was still increasing even if the practical actions for reducing phosphorus pollution load in the Basin of Lake Taihu had been taken. Meanwhile, the phosphorus content of Lake Taihu kept increasing since 2016, even if the exogenous phosphorus has been limited [10]. Therefore, it is speculated that the phosphorus released from the declination of bloom-forming cyanobacteria may also be a vital phosphorus source in the internal circulation, especially in the black patch events area.

### 3.2. Cyanobacterial decay in the dark accelerates dissolved inorganic phosphorus release

**Fig 3:**
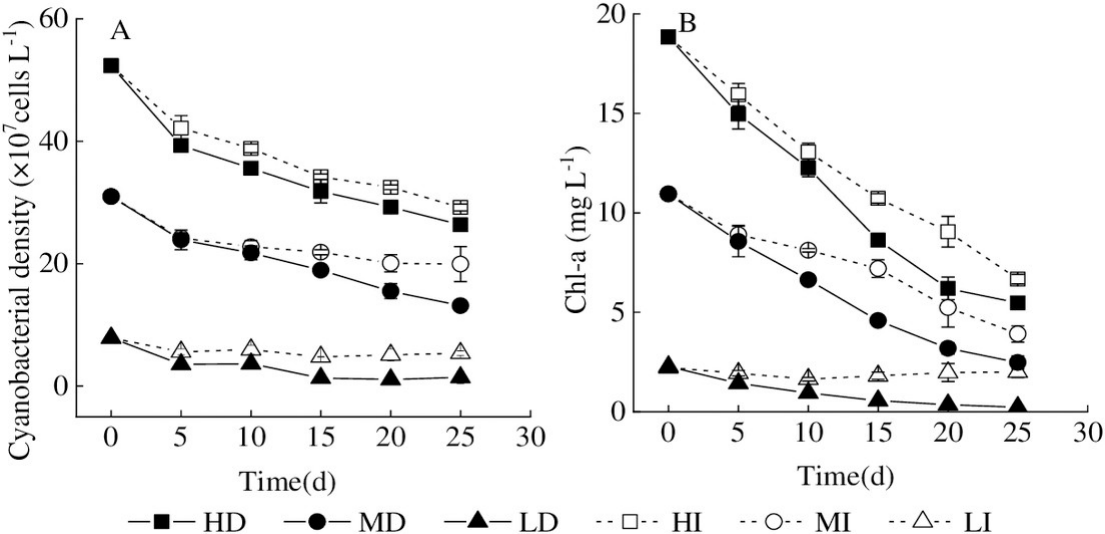
Variation of Chl-a concentration and accumulated cyanobacterial density in different treatments. Values given are the mean ± SD of three replicate measurements. A = cyanobacterial density, B = Chl-a.

Chl-a and cyanobacterial density are important biological indexes in the lake ecosystem, reflecting the intensity of water bloom [4, 11]. The present study showed that the net cyanobacterial mortality rates were 49.64%, 44.23%, 57.59%, 35.64%, 82.06%, and 31.58% in HD, HI, MD, MI, LD, and LI treatments respectively at the end of the experiment (Fig 3). Therefore, the gradual death of bloom-forming cyanobacteria coincided with the sustained declines of Chl-a and cyanobacterial cell density through the experimental period. Prior studies in Lake Dianchi had also noted that Chl-a content in enclosure decreased from 514 to 23.3 μg L^-1^ continuously when the algal bloom is dying out gradually in winter through the experimental period [11]. According to the results of Lake Taihu, Chl-a content and the cyanobacterial density maintained continuously decreasing in the intermediate and high cyanobacterial densities treatments [4]. However, the mortality rates in this study were far below Lake Dianchi (95.5% at the end of the experiment). On the one hand, the low temperature in winter determined the growth status of cyanobacteria in the enclosure of Lake Dianchi. On the other hand, cyanobacteria in the microcosm could decompose and utilize glycogen in cells in the form of fermentation to maintain life activities under high temperature and hypoxia conditions [35, 36].

According to the previous results [4, 11, 12, 14, 37], the P release from healthy metabolism and natural decay, could promote the occurrence of cyanobacterial blooms. There was no doubt that P release from the decline of bloom-forming cyanobacteria was the dominant process in this study because DTP and DIP contents in water were both significantly negatively correlated with cyanobacterial density and Chl-a, respectively (Table 2). In other words, aqueous DTP and DIP contents increased with the decline of cyanobacterial density and Chl-a. In addition, the cyanobacterial mortality rate is generally higher in the dark than that in the illumination, which may hint that cyanobacterial decomposition in the dark accelerates dissolved inorganic phosphorus release.

**Table 2:** Correlation between cell density and Chl-a with DTP and DIP.

	HD		MD		LD		HI		MI		LI	
	DTP	DIP	DTP	DIP	DTP	DIP	DTP	DIP	DTP	DIP	DTP	DIP
CD	-0.974*	-0.974*	-0.958*	-0.952*	-0.963*	-0.967*	-0.971*	-0.956*	-0.998*	-0.997*	-0.877	-0.885*
Chl-a	-0.923*	-0.931*	-0.942*	-0.939*	-0.978*	-0.997*	-0.912*	-0.892*	-0.902*	-0.905*	-0.591	-0.721

CD: cyanobacterial density.

*Means significant correlation.

Dark led to environmental stress on cyanobacteria, which may accelerate the decline and release of phosphorus from cyanobacteria. The current study found that the content of MDA and the activities of SOD and CAT in the dark were higher than in the illumination, especially in the low-density groups. Gao [38] reported that the reactive oxygen species (ROS) would be produced during metabolism under environmental stress, which could affect the normal metabolism of cells by increasing the permeability of the membrane and further destroying the structure of the cell membrane [39]. Antioxidant systems in cellular, including the antioxidant components (SOD, CAT) and non-enzymatic components (MDA), were stimulated to eliminate ROS and ensure the normal physiological metabolic function of cells [40, 41]. Thus, the contents of MDA and the activities of SOD and CAT of cyanobacteria were measured to monitor the physiological status. The MDA values in the dark treatments (MD, LD) were 8.85 times and 3.73 times that of the illumination group (MI, LI). CAT activity had a similar trend to those of MDA content. There was no significant difference in cyanobacterial SOD activity between the high accumulated density treatments and the medium cyanobacterial accumulated density treatments. However, both of the activities were significantly different from those of the low accumulated density group, resulting from that almost all cyanobacteria died at high and medium density.

**Fig 4:**
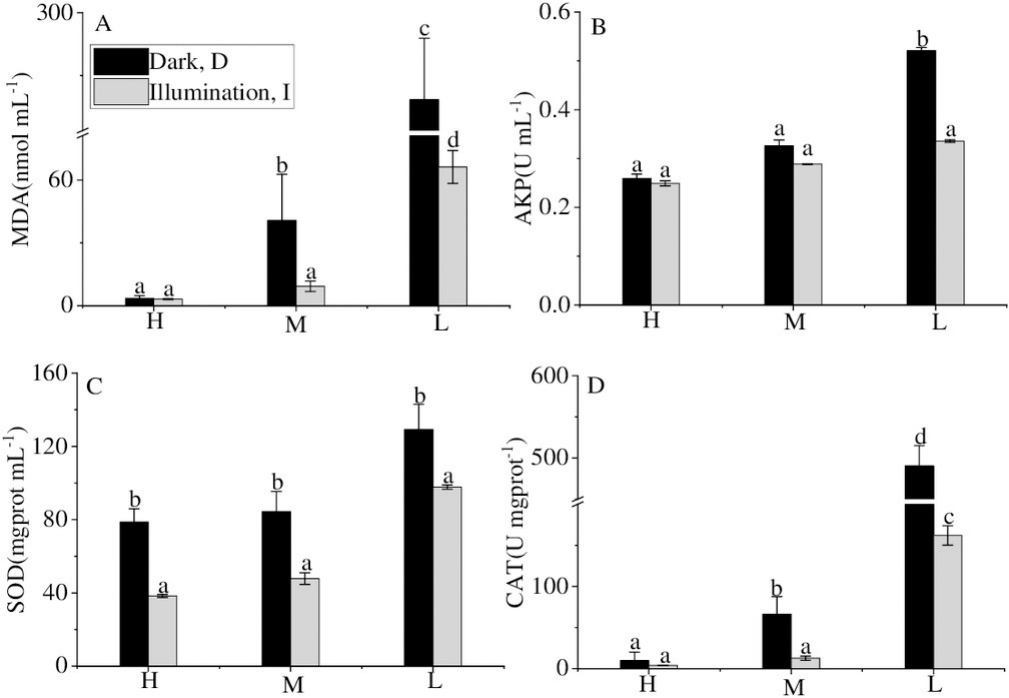
MDA, CAT content and SOD, AKP activity in different treatments at the end of the experiment. Values given are the mean ± SD of three replicate measurements. a, b, c and d indicate statistically significant differences from the controls, *P* < 0.05.

This research found that the AKP activity in the dark was higher than in the illumination condition (Fig 4B). Consistent with the previous study, Gao [38] showed that alkaline phosphatase (AKP) could hydrolyze monophosphate phosphate into orthophosphate. It was reported earlier that the AKP activity of cyanobacteria would increase in the dark [42]. Thus, dark significantly enhanced the activity of antioxidant systems within the cells and the content of alkaline phosphatase outside the cells at the same cyanobacterial density, which promoted cyanobacterial decay, which further induced the DIP release.

### 3.3 Changes in water quality with the cyanobacterial decomposition

**Fig 5:**
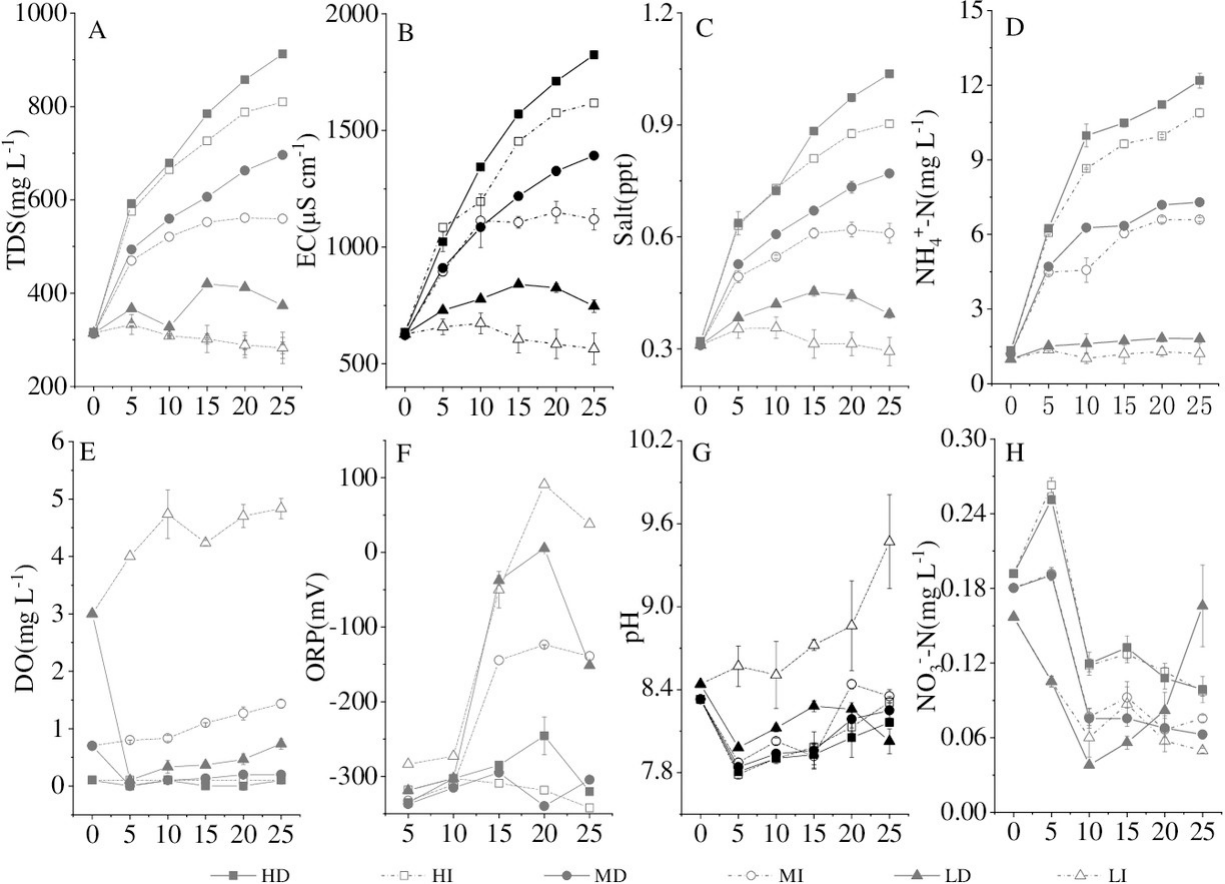
Variation of TDS (A), EC (B), Salt (C), NH_4_^+^-N (D), DO (E), ORP (F), pH (G) and NO_3_^-^-N(H) in different treatments, values given are the mean ± SD of three replicate measurements.

It has reported that the development and outbreak of cyanobacteria bloom could change the water quality [25], and the bloom-forming cyanobacterial decomposition was also accompanied by the changes in water parameters [4, 11]. In this declination experiment, TDS, EC, and Salt increased with the cyanobacterial decline in the medium and high densities (Fig 5A–5C), which was consistent with previous studies [4, 11, 24]. The water in the microcosm kept a strong reduction state in the medium and high cyanobacterial densities during the experiment and the lowest ORP value is -342.2 mV (Fig 5F). Simultaneously, the DO content in water fluctuated around 0 mg L^-1^ over the entire experimental period except in the low cyanobacterial density treatment (Fig 5E). The apparent decrement of DO and lower ORP were observed during the decomposition of bloom-forming cyanobacteria in microcosms with the medium and high (7.60×10^7^ cell L^-1^ and 8.85×10^8^ cell L^-1^) cyanobacterial densities [4]. The cyanobacterial decomposition process would increase pH value [11, 43]. The pH value in the present study fluctuated around 8, while the pH in the LI groups increased from 8.44 on the first day to 9.47 at the end of the experiment (Fig 5G). The difference may explain that a large amount of ammonia nitrogen (Fig 5D) was produced by cyanobacteria and nitrate-nitrogen reduced (Fig 5H) under the anoxic condition. Thus, the cyanobacterial decomposition would inevitably affect the physicochemical water parameters. In the inverse, the changes in the aqueous environment would accelerate the decomposition of bloom-cyanobacteria. Aqueous DTP and DIP contents increased with the increment of TDS, Salt, and EC under high and medium cyanobacterial densities (5.23×10^8^ cells L^-1^, 3.10×10^8^ cells L^-1^) (Table 3). Wu et al. [24] reported that anaerobic and strong reduction conditions accelerated the death and decomposition of cyanobacteria, promoting the P diffusion of cyanobacteria to the overlying water. Thus, the combined action of the bloom-forming cyanobacterial decomposition and its induced variation of the aqueous environment led to the increase of P release. Furthermore, at the same cyanobacterial density, dark significantly increased the TDS, EC, and Salt, and dramatically decreased ORP, DO and pH in water to accelerate DTP and DIP release during the decline of bloom-forming cyanobacteria.

**Table 3:** Correlation between the water quality parameters with DTP and DIP.

	HD		MD		LD		HI		MI		LI	
	DTP	DIP	DTP	DIP	DTP	DIP	DTP	DIP	DTP	DIP	DTP	DIP
TDS	0.967*	0.969*	0.988*	0.984*	0.688	0.722	0.993*	0.984*	0.994*	0.994*	-0.706	-0.554
Salt	0.955*	0.959*	0.989*	0.986*	0.803	0.830*	0.993*	0.985*	0.992*	0.995*	-0.208	-0.086
EC	0.956*	0.963*	0.972*	0.969*	0.795	0.828*	0.974*	0.964*	0.972*	0.969*	-0.485	-0.324
DO	-0.295	-0.281	-0.752	-0.754	-0.800	-0.750	0.876	0.760	0.812*	0.824*	0.930*	0.856*
pH	-0.464	-0.436	-0.268	-0.280	-0.583	-0.472	-0.230	-0.259	-0.006	0.004	0.679	0.532

### 3.4 Increase of specific bacteria promotes DIP release

**Fig 6:**
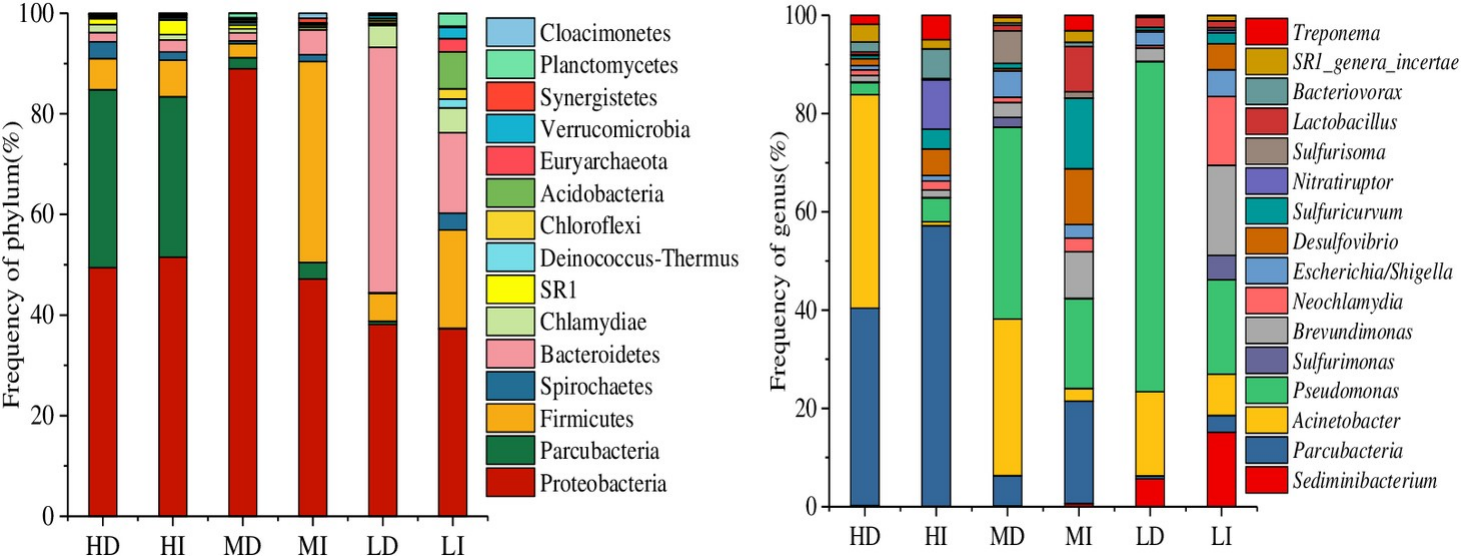
The abundance of bacterial phylum and genus accounts for the top 10% in each treatment.

Bacteria are one of the critical biologic regulators for the phytoplankton community structure. As mentioned in the previous study [44], the bacteria would increase rapidly and evolve into the dominant bacteria in the suitable nutrition water environment if the cyanobacteria decayed. Thus, 16S rDNA gene sequencing was used to analyze the bacterial community structures in the water at the end of the experiment when almost all cyanobacteria died. The main dominant bacterial phyla were Proteobacteria, Firmicutes, Bacteroidetes, Parcubacteria, and Actinobacteria (Fig 6). These five phylogenetic groups are common in Lake Taihu [45]. At low-density treatment (7.86×10^7^ cells L^-1^), dark significantly increased the abundance of Bacteroidetes strains, which are essential during cyanobacterial blooms [46] and can degrade not only macromolecular compounds [47], but also *Microcystis* cells [48]. At medium density treatment (3.10×10^8^ cells L^-1^), *Pseudomonas* and *Acinetobacter*, which belong to Proteobacteria that can decompose organic phosphorus, predominated in the dark [49]. High cyanobacterial density (5.23×10^8^ cells L^-1^) picked Parcubacteria and Proteobacteria to be dominant in the water, and the amounts of highly efficient organophosphorus degrading bacteria (*Acinetobacter*) in Proteobacteria increased in the dark. Overall, dark promoted organic matter degrading bacteria and organic phosphorus degrading bacteria absolute predominance in the decay of accumulated bloom-forming cyanobacteria.

The number of OTUs, the Shannon, Chao1, and Simpson index were calculated for the bacterial samples. The highest bacterial OTUs number (3783) occurred at the low cyanobacterial accumulated density with light (LI). While the lowest number of bacterial OTUs appeared at high accumulated cyanobacterial density in the dark (HD). Shannon index is sorted as follows: LI > LD, MI > MD, HI > HD. Simpson index was in the order of HD > HI, MD > MI, LD > LI (Table 4). This finding was consistent with the detection results in the previous studies of the decomposition of cyanobacterial blooms [33, 44, 49, 50]. It was interesting to found that high accumulated cyanobacterial density markedly reduced bacterial diversity in the dark. Therefore, the decrease of bacterial diversity and the preponderance of organic matter degrading bacteria and organic phosphorus degrading bacteria accelerate the DTP release from the decay of accumulated bloom-forming cyanobacteria in the dark, which may be a crucial factor for the continuous outbreak of cyanobacterial bloom.

**Table 4:** Alpha-diversity index analysis.

Sample	OTUs	Shannon index	Chao1 index	Simpson index
HD	1234	3.61	1413.69	0.13
HI	1437	3.95	1563.50	0.12
MD	1472	3.73	1700.30	0.10
MI	1520	4.20	1655.45	0.05
LD	1691	5.15	1755.26	0.02
LI	3783	6.64	3877.77	0.01

## 4 Conclusion

A microcosm experiment was set up to study P release from accumulated bloom-forming cyanobacteria in the dark. Our results demonstrated that dark status markedly increased the proportion of DIP in water during the decline of bloom-forming cyanobacteria. The DTP and DIP release from cyanobacteria became greater when high accumulated cyanobacterial density appeared in the dark. Besides, P release of bloom-forming cyanobacteria was a result of cyanobacterial apoptosis which would affect the physicochemical water parameters. In the inverse, the changes in the aquatic environment would accelerate the decomposition of bloom-forming cyanobacteria. Moreover, the DIP release from high-density cyanobacteria (7.86×10^7^ cells L^-1^~5.23×10^8^ cells L^-1^) resulted from the relative abundance of organophosphorus degrading bacteria in the dark. Therefore, DIP released from accumulated cyanobacterial blooms may reveal the reasons for the temporal and spatial differences of phosphorus distribution in Lake Taihu, especially in the black patch events area. Furthermore, the phosphorus release of cyanobacteria provides the nutrient for the new round of cyanobacteria bloom, which may explain the continuous outbreak of cyanobacterial bloom. Therefore, the study of phosphorus release from cyanobacteria may provide a theoretical basis for the control of cyanobacteria and a solution to improve water environment quality.

## Supporting information

S1 Table Water quality parameters in different water layers at the sampling site. (DOCX)

S2 Table Water quality parameters in upper and 10 cm below the surface. (DOCX)

S1 Fig The wind speed and direction of Lake Taihu in summer among 2011–2018. (DOCX)

S2 Fig Variation of DIP, DTP, NO_3_^-^-N, NO_2_^-^-N, NH_4_^+^-N in surface and lower water of in situ experiments, UP = surface layer, BELOW = 10 cm below the surface. Values given are the mean±SD of three replicate measurements. Asterisks indicate statistically significant differences from the controls; * *P*< 0.05.
(DOCX)

S3 Fig Cyanobacterial accumulation area in the lake bay of Lake Taihu in summer. (DOCX)

S4 Fig Cyanobacterial accumulation area in the lake bay of Lake Taihu in winter. (DOCX)
