# Supplementary material for: Dark accelerates dissolved inorganic phosphorus release of high-density cyanobacteria
Source: PLoS One. 2020 Dec 22;15(12):e0243582. doi: 10.1371/journal.pone.0243582 (PMC7755282; doi:10.1371/journal.pone.0243582)
Supplement: S1 Table — (DOCX) [file pone.0243582.s001.docx]

Table S1 Water quality parameters in different water layers at the sampling site

| Parameters | The surface layer | 5cm below the  surface layer |
| --- | --- | --- |
| Water depth (cm) | 110 |  |
| Temperature (℃) | 30.0 | 29.9 |
| DO (mg L^-1^) | 8.07 | 0.63 |
| pH | 8.72 | 7.62 |
| ORP (mV) | 100.7 | -132.3 |
| Cond (μs cm^-1^) | 860 | 871 |
| TDS (mg L^-1^) | 410 | 438 |
