# Supplementary material for: Dark accelerates dissolved inorganic phosphorus release of high-density cyanobacteria
Source: PLoS One. 2020 Dec 22;15(12):e0243582. doi: 10.1371/journal.pone.0243582 (PMC7755282; doi:10.1371/journal.pone.0243582)
Supplement: S2 Table — (DOCX) [file pone.0243582.s002.docx]

Table S2. Water quality parameters in upper and 10 cm below the surface

| Parameters | Eh  (mV) | pH | DO  (mg L^-1^) | Cond  (us cm^-1^) | SALT  (ppt) | TDS  (mg L^-1^) |
| --- | --- | --- | --- | --- | --- | --- |
| UP | -35.2 | 7.22 | 1.91 | 860 | 0.47 | 422 |
| BELOW | -60.7 | 7.46 | 0.75 | 871 | 0.73 | 438 |
