# Supplementary figures and images for: Dark accelerates dissolved inorganic phosphorus release of high-density cyanobacteria
Source: PLoS One. 2020 Dec 22;15(12):e0243582. doi: 10.1371/journal.pone.0243582 (PMC7755282; doi:10.1371/journal.pone.0243582)

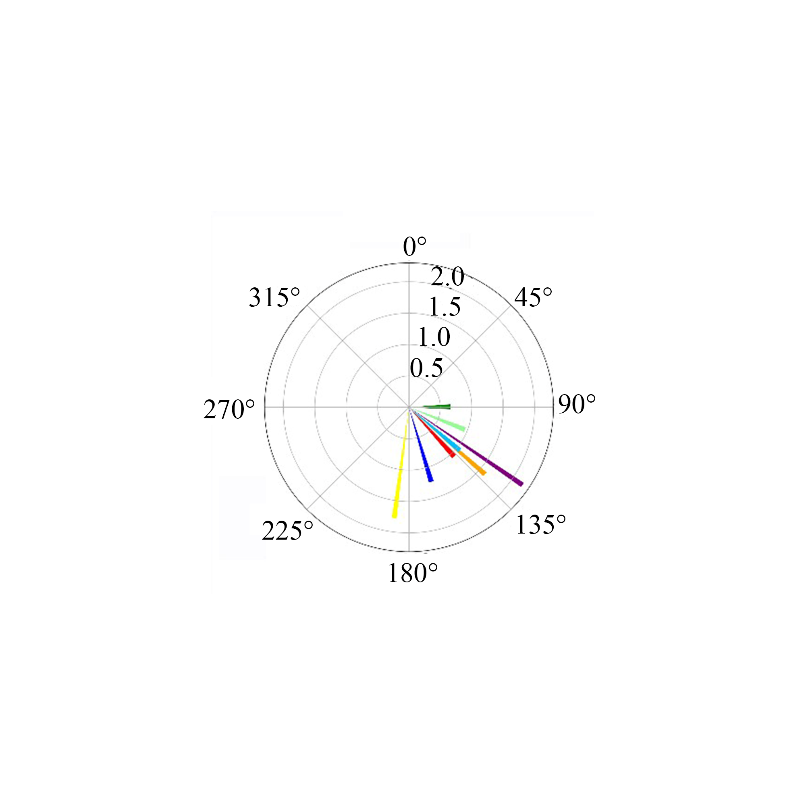


Fig. S1 The wind speed and direction of Lake Taihu in summer among 2011-2018

Supplement: S1 Fig — (DOCX) [file pone.0243582.s003.docx]

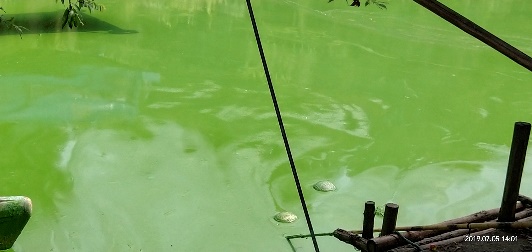

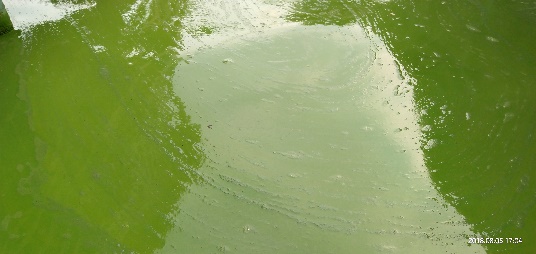


Fig. S3 Cyanobacterial accumulation area in the lake bay of Lake Taihu in summer

Supplement: S3 Fig — (DOCX) [file pone.0243582.s005.docx]

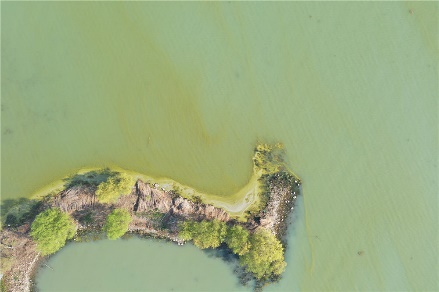

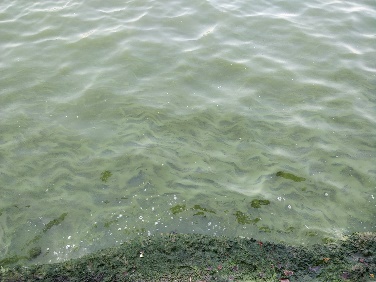


Fig. S4 Cyanobacterial accumulation area in the lake bay of Lake Taihu in winter

Supplement: S4 Fig — (DOCX) [file pone.0243582.s006.docx]
