# Supplementary material for: Dark accelerates dissolved inorganic phosphorus release of high-density cyanobacteria
Source: PLoS One. 2020 Dec 22;15(12):e0243582. doi: 10.1371/journal.pone.0243582 (PMC7755282; doi:10.1371/journal.pone.0243582)
Supplement: S2 Fig — Values given are the mean±SD of three replicate measurements. Asterisks indicate statistically significant differences from the controls; * P< 0.05. (DOCX) [file pone.0243582.s004.docx]

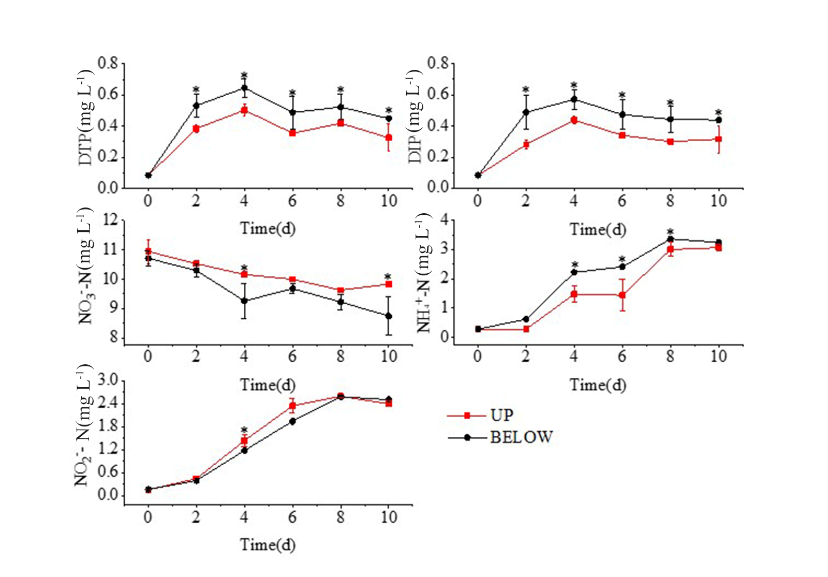


Fig. S2 Variation of DIP, DTP, NO_3_^-^-N, NO_2_^-^-N, NH_4_^+^-N in surface and lower water of in situ experiments, UP = surface layer, BELOW = 10 cm below the surface. Values given are the mean±SD of three replicate measurements. Asterisks indicate statistically significant differences from the controls; * *P*< 0.05.
